# Dose-Dependent Effects of Minocycline on Facial Nerve Regeneration: A Microscopic and Molecular Study

**DOI:** 10.1007/s00266-026-05976-z

**Published:** 2026-08-06

**Authors:** Sumera İnci Yılmaz, Sedat Meydan, Mukaddes Eşrefoğlu, Nihat Mustafayev, Leyla Mustafayeva, Kübra Başol Baki, Metin Demirel, Fatih Maral, Şahabettin Selek

**Affiliations:** 1 Faculty of Medicine, Bezmialem Vakıf University, Istanbul, Turkey; 2 Faculty of Medicine, Department of Anatomy, Bezmialem Vakıf University, Istanbul, Turkey; 3 Faculty of Medicine, Department of Histology and Embryology, Bezmialem Vakıf University, Istanbul, Turkey; 4 Nihat Mustafayev Clinic, Istanbul, Turkey; 5 Faculty of Medicine, Department of Physical Therapy and Rehabilitation, Cerrahpaşa Istanbul University, Istanbul, Turkey; 6 Faculty of Medicine, Department of Medical Biochemistry, Bezmialem Vakıf University, Istanbul, Turkey; 7 Faculty of Medicine, Department of Anatomy, Marmara University, Istanbul, Turkey; 8 Department of Medical Services and Techniques, Vocational School of Health Services, Pathology Laboratory Techniques Program, Bezmialem Vakıf University, Istanbul, Turkey

**Keywords:** Electrophysiology, LC-MS/MS, Microscopy, Minocycline, Nerve regeneration

## Abstract

Injuries to the facial nerve present clinical difficulties with frequently unfavorable results. This study uses histological, electrophysiological, and LC-MS/MS-based metabolomic techniques to examine the dose-dependent effects of locally administered minocycline on facial nerve regeneration. For that purpose, the rats were divided into three groups. Following nerve transection and suturing, the rats from Group A received sterile water, those from Group B received 40 μg of minocycline, and those from Group C received 100 μg of minocycline locally on the suture line in 20 μL fibrin gel. Nerve regeneration was evaluated using LC-MS/MS metabolomics and histopathological analyses at the end of the 28th day. Electrophysiological analyses were performed on the 0th day and postoperative 1, 2, and 4 weeks. In the sections of Group A, edema within the myelin sheath and beneath the perineurium were the most conspicuous findings. Vacuolar characteristics within the myelin sheath were also detectable. Groups B and C exhibited preserved architecture with minimal edema. Nerve fiber density was similar across groups (*p* > 0.05). Metabolomics analyses revealed reduced betaine and elevated proline levels in Group C vs. Group A (*p* < 0.05). Compound muscle action potential values improved over time, especially in Group B (*p* = 0.013), peaking at week 2. Both doses of minocycline have been found beneficial on facial nerve regeneration. Although Group C showed superior histological and biochemical outcomes, Group B induced greater clinical and electrophysiological outcomes. We suggest that minocycline is effective on nerve regeneration in a dose-dependent manner.

*No Level Assigned* This journal requires that authors assign a level of evidence to each submission to which Evidence-Based Medicine rankings are applicable. This excludes Review Articles, Book Reviews, and manuscripts that concern Basic Science, Animal Studies, Cadaver Studies, and Experimental Studies. For a full description of these Evidence-Based Medicine ratings, please refer to the Table of Contents or the online Instructions to Authors www.springer.com/00266 .

## Introduction

Physical trauma from car crashes, violence, sports injuries, and iatrogenic causes frequently results in severe nerve damage [1–3]. These injuries may result in physical handicap and loss of motor and sensory abilities, which will lower the patient’s quality of life [4]. Depending on how serious the injury is, there are a variety of treatment choices, such as physical rehabilitation, surgery, and pain medication. However, results can be unpredictable, and recuperation is frequently drawn out.

The gold standard nerve autograft transfer [5,6], primary nerve repair, and conduit-assisted repair methods [7] are surgical options for treating peripheral nerve damage. Analgesics and corticosteroids are frequently used in non-surgical treatment to manage pain, and electrical nerve stimulation can help promote functional recovery [8].

The regenerative effects of substances such as bumetanide and dexamethasone on the facial nerve [9], as well as insulin-like growth factor-1 (IGF-1) and platelet-rich plasma (PRP) [10], have been studied in earlier clinical and experimental investigations. Minocycline’s antibacterial [11], anti-inflammatory [12], neuroregenerative [13–15], and neuroprotective [16] qualities have made it an attractive therapeutic choice among medicinal drugs. While its regenerative effects on the sciatic nerve have been previously documented [1], no experimental studies have examined the potential regenerative effects of its local application on the facial nerve. This study aimed to examine the cellular, molecular, and functional outcomes by histological examination, electro-neurophysiological testing, and metabolomic analysis in order to determine the dose-dependent effects of locally administered minocycline on facial nerve regeneration.

Liquid chromatography-mass spectrometry (LC-MS), a highly sensitive and specific technique, can be applied to a wide range of biological molecules and can be used in clinical biochemistry [2]. Recent developments in LC-MS contributed to its rising popularity by making biomarker discovery and precision medicine easier [3]. For instance, a study using LC-MS to analyze the rat cochlear nucleus at different developmental stages identified significant metabolomic differences related to maturation [4]. Another study using LC-MS in a rodent neuropathic pain model revealed significant changes in dorsal root ganglion and serum metabolites [5]. In our study, LC-MS analysis was performed to investigate the metabolomic impact of minocycline following facial nerve injury in rats. To our knowledge, this is the first study to integrate functional, cellular, and metabolomic evaluations to assess the effects of minocycline in this context.

## Materials & Methods

### Animals and Experimental Model

**Fig. 1:**
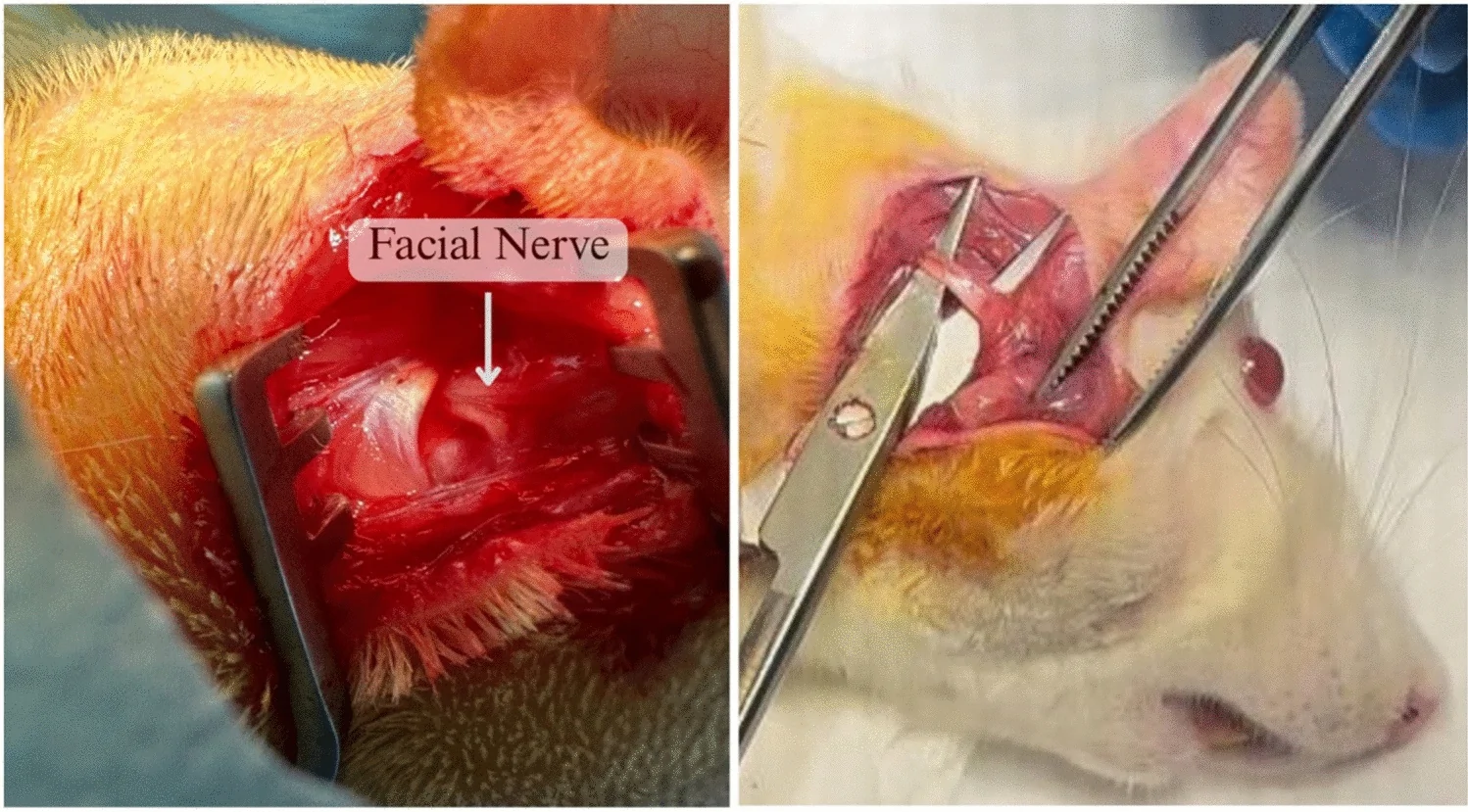
Facial nerve identified behind the digastric muscle. The facial nerve was exposed, transected, and sutured

In our study, 26 male Wistar Albino rats weighing 250–300g were used. The research was conducted in accordance with the principles of the 1964 Helsinki Declaration and was approved by the ethics committee of the Experimental Application and Research Center, Bezmialem Vakif University (No: 2023-60). Animals had free access to standard rat chow and water at controlled room temperature (22–25 °C) and humidity on a 12-h light/dark cycle for the duration of the study. The rats were divided into three randomized groups as follows (Table 1): Group A (control): The rats received sterile water locally on the suture line in 20 μL fibrin gel; Group B: The rats received 40 μg of minocycline locally on the suture line in 20 μL fibrin gel; Group C: The rats received 100 μg of minocycline locally on the suture line in 20 μL fibrin gel. For all surgical procedures, the right side of the face was first shaved and sterilized with povidone-iodine. Then, a 1-cm long oblique incision was made below the helix of the ear, carefully preserving the parotid gland and preauricular lymph node. The cartilage of the external ear canal was exposed, and the retromandibular vein was observed allowing visualization of the main branches of the facial nerve. The two primary branches of the facial nerve were transected, and nerve anastomosis was achieved by suturing separated ends with 8/0 Prolene mattress sutures. The skin was closed using 5/0 Prolene, and the wound area was properly sterilized. Body weight for each rat was recorded weekly. At the end of the 28-day experimental period, all rats were euthanized by decapitation. The facial nerve segment at the anastomosis site, marked by the suture, was carefully excised from each rat. The harvested tissues were then prepared for histological and metabolomic analyses (Fig. 1).

**Table 1:** Description of the treatment conditions administered locally to the reanastomosed facial nerve across the study groups

Group	Agent and dose	Administration method
A (Control)	Sterile water in 20 µL of fibrin gel	Delivered locally on the suture line of the reanastomosed facial nerve
B	40 µg of minocycline in 20 µL of fibrin gel	Delivered locally on the suture line of the reanastomosed facial nerve
C	100 µg of minocycline in 20 µL of fibrin gel	Delivered locally on the suture line of the reanastomosed facial nerve

### Histological Examination

Following fixation in 10% neutral buffered formaldehyde, tissues underwent dehydration with alcohol and clearance with xylene before being embedded in paraffin. Subsequently, 5-μm sections were obtained and stained with hematoxylin-eosin (H&E), methylene blue, and modified Gomori’s techniques for microscopic evaluation. Image-J software (version 1.53) was used to calculate the intensity of the silver staining as a marker of content of nerve fibers. Percentage area analyses of three successive fields per section at ×40 magnification were conducted to evaluate positive staining. Samples were evaluated under a Nikon light microscope (Eclipse 920248, USA), and the images were transferred to a computer using a fully compatible imaging software connected to a Nikon DS-Fi2-U3 camera.

### Metabolomics Analysis

A part of the harvested tissue samples was stored at −86 °C until metabolomics analyses. Prior to extraction, samples were thawed on ice and 100 mg of nerve tissue was accurately weighed. A volume of 1 mL of 60 mM phosphate buffer (pH 7.4) was added, and the mixtures were homogenized using a bead-based homogenizer at 30 Hz for 10 min. The homogenates were then centrifuged at 20,000 g for 10 min at 4 °C. The resulting supernatants were filtered through 0.22 μm cellulose acetate syringe filters, and 100 μL of the clarified extract was transferred into polypropylene vials for analysis.

Untargeted metabolite profiling was performed at the Bezmialem Vakıf University Drug Research Center (İLMER) using a Q Exactive™ Plus Hybrid Quadrupole-Orbitrap™ mass spectrometer (Thermo Fisher Scientific, Germany) coupled to a UHPLC system. Chromatographic separation was achieved on a Fortis C18 column (150 × 2.0 mm, 3 μm particle size). The mobile phases consisted of water containing 0.1% (v/v) formic acid (mobile phase A) and methanol (mobile phase B). The gradient elution program was as follows: Mobile phase B was increased linearly from 5% to 95% over the first 18 min and then returned to the initial conditions, giving a total analytical run time of 20 min. The flow rate was 0.3 mL/min, the column temperature was maintained at 40 °C, and the injection volume was 5 μL.

To monitor analytical stability and correct for potential signal drift, a pooled quality control (QC) sample was prepared by combining equal volumes of each study sample. The injection sequence was randomized, and QC samples were injected at the beginning of the run for column conditioning and subsequently after every 10 study samples. Procedural blanks (extraction solvent without tissue) were also analyzed to identify background and carry-over features. System performance was considered acceptable when the relative standard deviation (RSD) of QC peak areas was ≤ 30% for the majority of detected features, and retention time shifts remained within ± 0.1 min across the batch.

The Q Exactive™ Plus mass spectrometer was operated in heated electrospray ionization (HESI) mode in both positive and negative polarities. Typical source settings were as follows: spray voltage ~3.5 kV, capillary temperature ~ 320 °C, sheath and auxiliary gas values set according to the manufacturer’s recommendations, and the S-lens RF level optimized daily using a calibration solution. Full MS spectra were acquired over an *m*/*z* range of 70–1000. Raw data files were converted to mzML and MGF formats using ProteoWizard (peak-picking enabled, centroid mode). Data pre-processing, including peak detection, deconvolution, alignment, and gap filling, was carried out in MZmine 3.0. Subsequent statistical analysis and metabolite annotation were performed using the TidyMass R package and MetaboAnalystR, and metabolite identification was supported by spectral matching against the HMDB, METLIN, and MoNA mass spectral databases.

### Electrophysiological Assessment

**Fig. 2:**
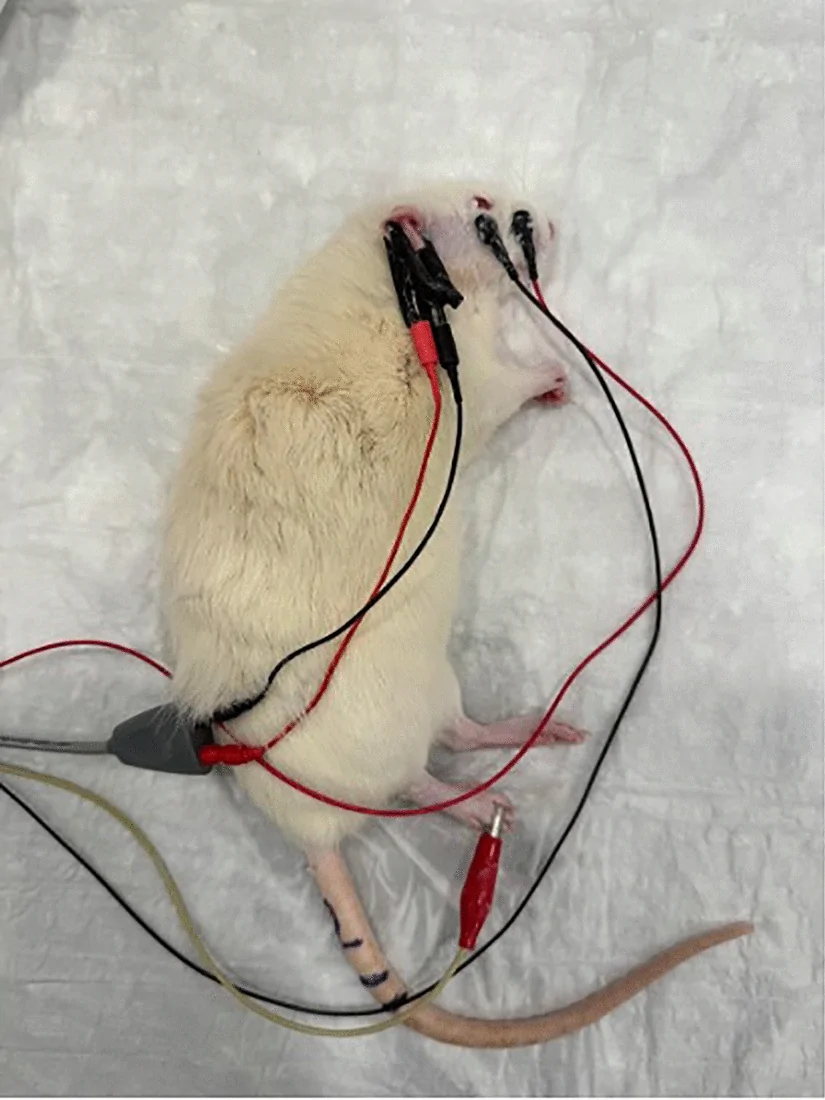
Electrophysiological analysis conducted on rats

A comprehensive electrophysiological testing protocol was implemented to evaluate nerve regeneration following facial nerve injury. Electromyography (EMG) recordings were conducted using the Neuron Spectrum-5 device, ensuring accurate measurement of nerve conduction and muscle responses. The assessments were performed by an experienced neurology specialist in electro-neurophysiology, focusing on key parameters such as distal motor latency (DML) and compound muscle action potential (CMAP) values. Baseline nerve conduction was assessed bilaterally in rats prior to the procedure, revealing comparable values on both sides, thus providing a reliable control for post-injury comparisons. Measurements were conducted at four time points: baseline (0W), 1st week (1W), 2nd week (2W), and 4th week (4W), following the achievement of supramaximal stimulation to ensure consistent and reproducible results. All nerve conduction procedures were performed under deep anesthesia to minimize discomfort and eliminate motion artifacts that could interfere with the accuracy of the measurements. Stimulation parameters were meticulously standardized, with an intensity of 2.0 mA to guarantee supramaximal stimulation, a pulse duration of 100 ms, and an inter-stimulus interval of 20 ms. The polarity was set to positive to maintain consistent nerve activation throughout the assessments. Electrode placement was crucial for the accuracy of the measurements. Stimulating electrodes were positioned near the auricular region; while recording and reference electrodes were placed subcutaneously on the right side of the orbicularis oris muscle to capture CMAPs effectively. A ground electrode was placed on the toe to minimize electrical noise and enhance signal clarity (Fig. 2).

### Statistical Methods

For the statistical analyses of the evaluation of histopathological results and the electrophysiological results, IBM SPSS Statistics version 26 was used. For the variables to be measured in the study, when a mean difference of 0.28 units between groups (means: 0.23 and 0.51; standard deviations: 0.08 and 0.21) was considered significant, the required sample size was calculated as nine subjects per group with 80% power at a 95% confidence level. A total of 27 male rats will be used in the study. For the histopathological analyses, Mann–Whitney U-test was used for comparing the medians of two independent groups. The descriptive statistics for the quantitative variables in the study were provided as mean, median, standard deviation, minimum, and maximum. A statistical significance level of 0.05 was adopted.

For the statistical analyses of the electrophysiological results, one-way ANOVA was utilized to assess differences between groups, particularly when the data followed a normal distribution. This test helped determine whether there were any statistically significant differences in the measured parameters, such as CMAP and DML values, across the different treatment groups. For data that did not meet the assumptions of normality, the non-parametric Friedman test was employed. This test is well-suited for analyzing repeated measures data within groups, allowing for the detection of changes over time without relying on the normal distribution of the data. Repeated measures ANOVA were conducted to evaluate longitudinal data, enabling the assessment of within-subject variability over different time points. This analysis helped identify trends in nerve regeneration and the potential effects of minocycline treatment across various time intervals. To further explore specific differences between time points, Wilcoxon signed-rank tests were performed for pairwise comparisons. This non-parametric test is ideal for comparing related samples, particularly when dealing with small sample sizes or non-normally distributed data. Bonferroni correction was applied to adjust for multiple comparisons, reducing the risk of Type I errors and ensuring the robustness of the statistical conclusions.

For LC-MS/MS analyses, raw LC–MS data were processed to generate a feature table containing the mass-to-charge ratio (m/z), retention time (RT), and corresponding peak intensities across all samples. Following pre-processing with MZmine 3.0 (peak detection, deconvolution, alignment, and gap filling), the resulting peak intensity matrix was imported into R for further analysis using the TidyMass and MetaboAnalystR packages. Features with more than 20% missing values in at least one experimental group and those with a QC RSD >30% were removed to ensure analytical robustness. Remaining peak intensities were log₁₀-transformed to reduce heteroscedasticity and then auto-scaled (mean-centered and divided by the standard deviation) prior to multivariate analysis. When required, batch effects were evaluated and corrected using QC-based signal correction algorithms implemented in the TidyMass environment. Residual missing values were imputed using k-nearest neighbor (kNN) imputation.

Metabolite annotation was performed by matching accurate mass, retention time, and MS/MS spectra against public libraries (HMDB, METLIN, and MoNA) within predefined tolerances. Putatively identified metabolites were reported according to the Metabolomics Standards Initiative (MSI) at level 2. For each identified metabolite, an overall identification confidence score was automatically calculated by the TidyMass package, integrating m/z match, retention time match, and MS^1^–MS^2^ spectral similarity metrics.

Unsupervised multivariate analyses, including principal component analysis (PCA) and hierarchical clustering, were conducted to explore intrinsic variation and sample distribution. For class discrimination, partial least squares discriminant analysis (PLS-DA) and orthogonal PLS-DA (OPLS-DA) models were constructed, and model performance was evaluated using cross-validation and permutation testing. To identify significantly altered metabolites between groups, appropriate univariate statistical tests were applied based on data distribution: Student’s *t*-test for normally distributed data and the Mann–Whitney *U*-test for non-normally distributed data. Resulting p-values were adjusted for multiple comparisons using the Benjamini–Hochberg false discovery rate (FDR) method. Metabolites were considered statistically significant if they exhibited an adjusted *p*-value < 0.05 and an absolute fold change (|FC|) greater than 1.5. Differential features were visualized using volcano plots and heatmaps, and the discriminatory performance of key metabolites was further evaluated by receiver operating characteristic (ROC) curve analysis. Pathway and metabolite set enrichment analyses were performed using the KEGG and SMPDB libraries integrated within the MetaboAnalystR platform.

## Results

### Clinical changes

**Fig. 3:**
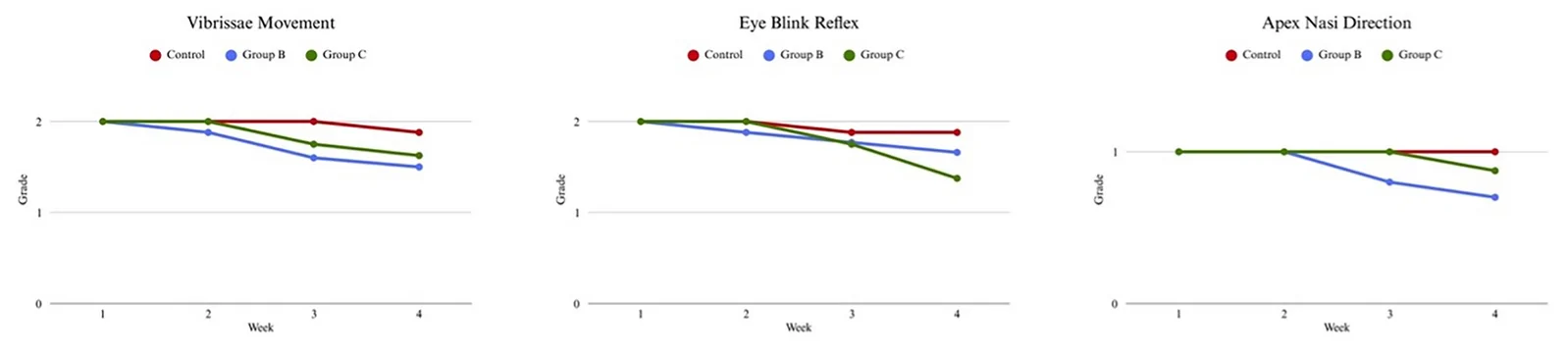
Weekly prevalence of clinical signs in each treatment group (Group B: 40 µg minocycline and Group C: 100 µg minocycline). Clinical scores were defined as follows: 2 = baseline after injury (no function), 1 = partial recovery in vibrissae movement and eye-blink reflex with skewed apex nasi, and 0 = complete recovery. A greater proportion of rats demonstrated functional improvement in Group B compared with Groups A and C

Clinical changes were observed and documented in each group preoperatively and postoperatively at the end of the 1st, 2nd, and 4th weeks. Facial nerve paralysis is assessed by observing a skewed apex nasi, weak vibrissae movement, and the inability to blink the eye on the affected side of the face. In Group B, improvement in facial nerve functions was more noticeable compared to Groups A and C (Fig. 3).

**Fig. 4:**
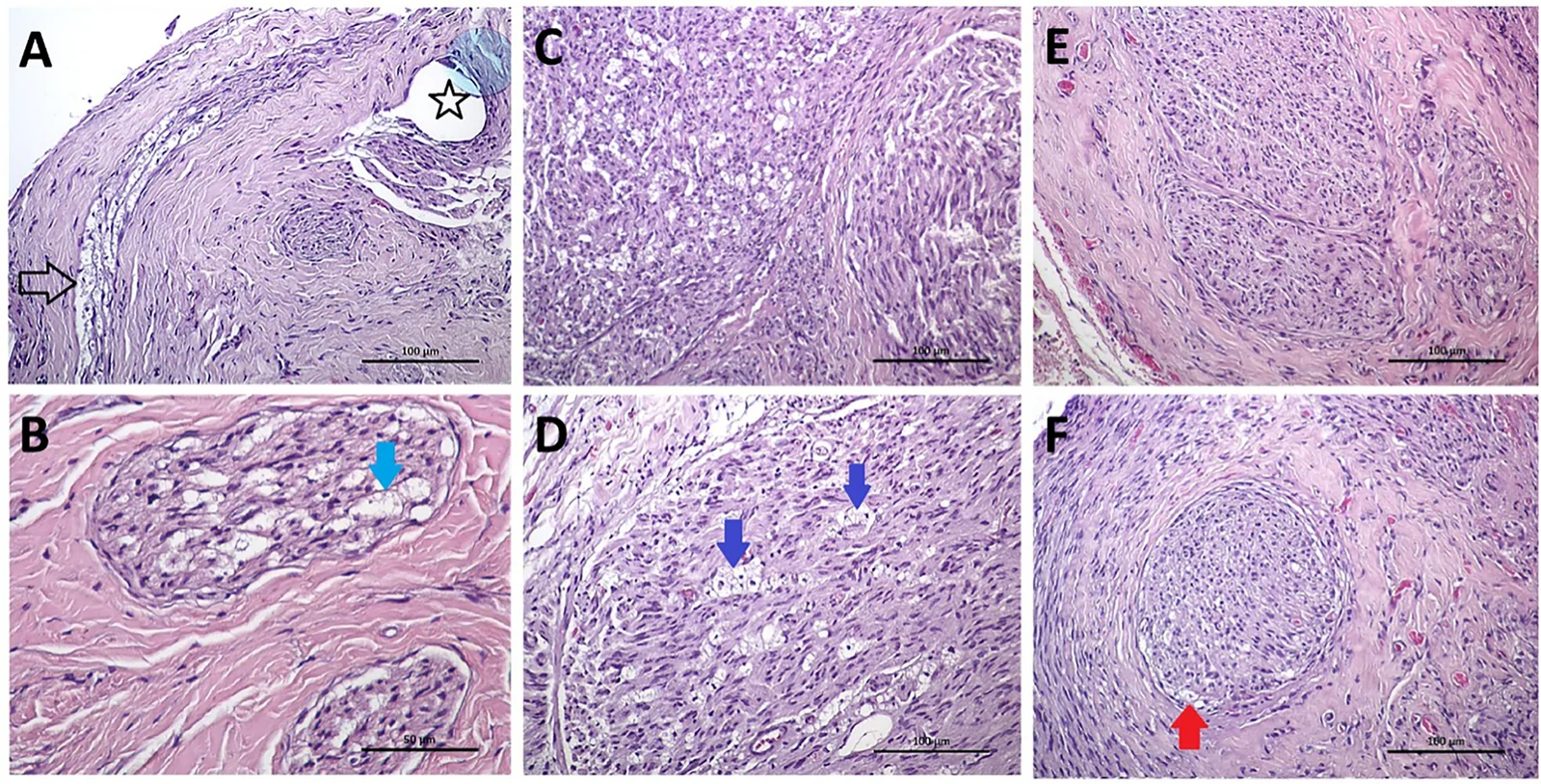
**A** In a section from Group A, edema is marked with an arrow. The asterisk shows the surgical suture point. H-E; X 20. **B** In a section from Group A, the cross-section of the nerve fiber shows signs of edema. The diameter of the nerve fibers is not uniform as a sign of edema within the myelin sheath. Besides vacuoles in different sizes are observed within the myelin sheath (blue arrow). H-E; X 40. **C** In a section of Group B, the nerve fiber on the left shows some signs of minimal edema within the myelin sheath. H-E; X 20. **D** In a section of Group B, rarely some vacuoles (blue arrows) are seen within the myelin sheath. H-E; X 20. **E** In a section of Group C, no signs of edema were detected. The tissue is intact and healthy. H-E; X 20. **F** In a section of Group C, no signs of edema were detected (E). The tissue is intact and healthy. In a limited area, edema within the myelin sheath is observed (red arrow). H-E; X 20

**Fig. 5:**
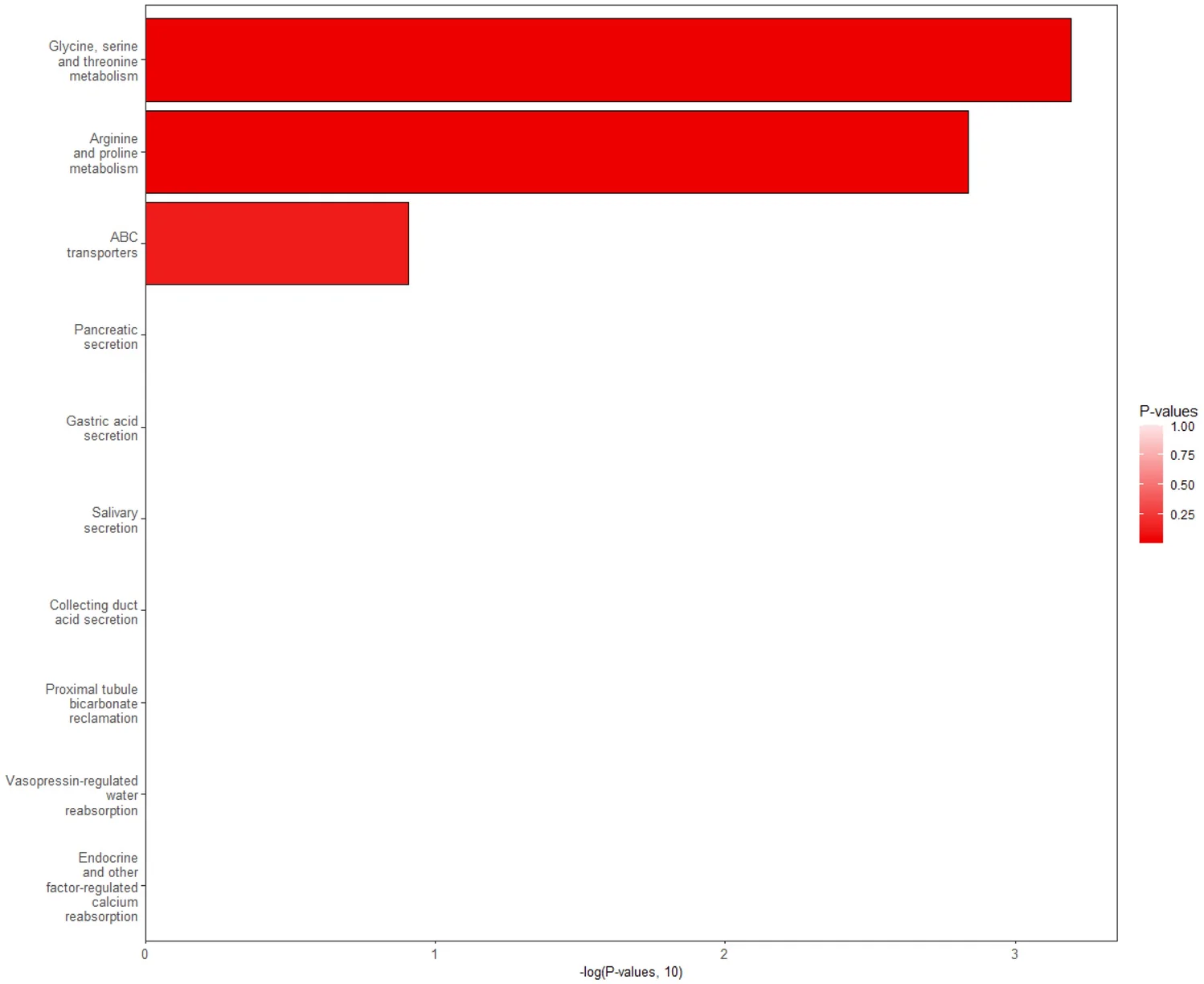
Pathway analysis of A vs. B groups (glycine, serine, and threonine metabolism *p* = 0.001, arginine and proline metabolism *p* = 0.002, and ABC transporters *p* = 0.063)

Histopathological results: The most conspicuous finding detected in the sections of Group A was edema (Fig. 4A, B). In cross-section, the diameter of the nerve fibers was not uniform probably because of the edema within the myelin sheath (Fig. 4B, C). Edema was also prominent just beneath the perineurium (Fig. 4C). Vacuolar characteristics within the myelin sheath were also detectable (Fig. 4B, C). The histological features of facial nerve and perineuronal tissue were pretty good in the sections of Group B. Generally, no signs of edema were detected except minimal vacuolar appearance within the myelin sheath (Figs. 4D, 5E). Similar to Group C, the histological features of facial nerve and perineuronal tissue were pretty good. In the sections of this group, edema was not detected in a few sections rarely edema within the myelin sheath was observed (Fig. 4F). Nerve fiber intensities of Groups A, B, and C were 29 ± 1.22, 29.60 ± 1.63, and 29.42 ± 1.48; respectively. No statistically significant difference was observed in terms of nerve fiber density between the groups (*P* > 0.05).

### LC-MS/MS results

**Fig. 6:**
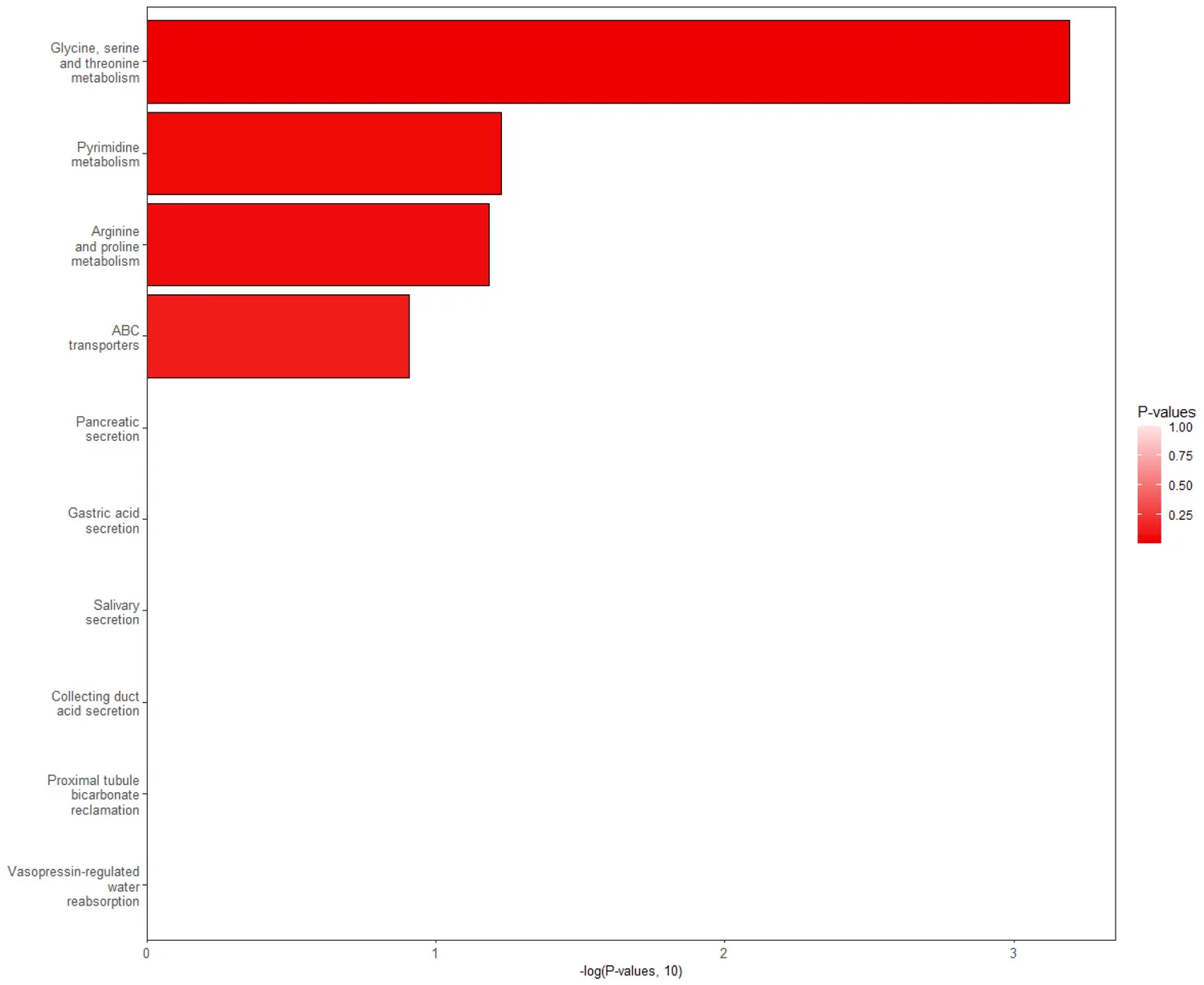
Pathway analysis of A vs. C groups (glycine, serine, and threonine metabolism *p* ≈ 0.00063, pyrimidine metabolism *p* ≈ 0.0079, arginine and proline metabolism *p* ≈ 0.010, and ABC transporters *p* ≈ 0.050)

**Fig. 7:**
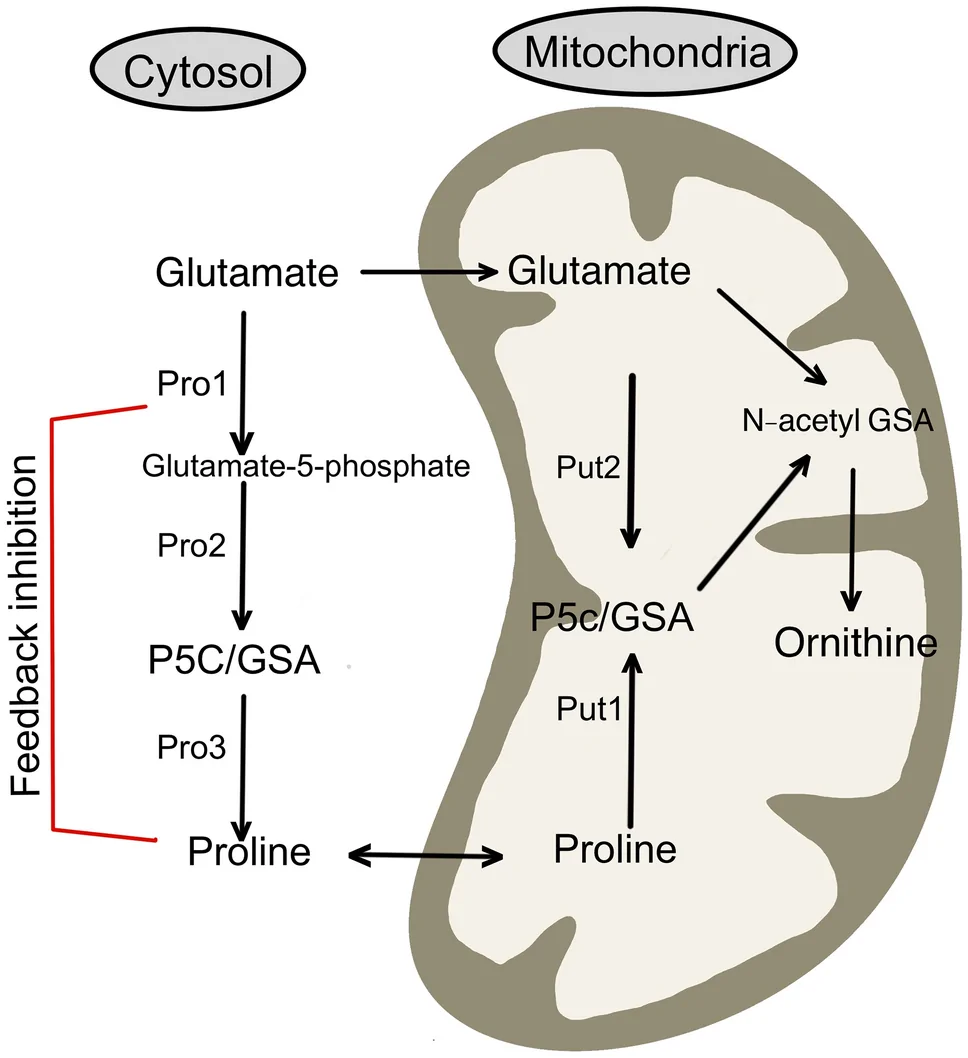
Metabolic pathway of proline in *Saccharomyces cerevisiae*

**Fig. 8:**
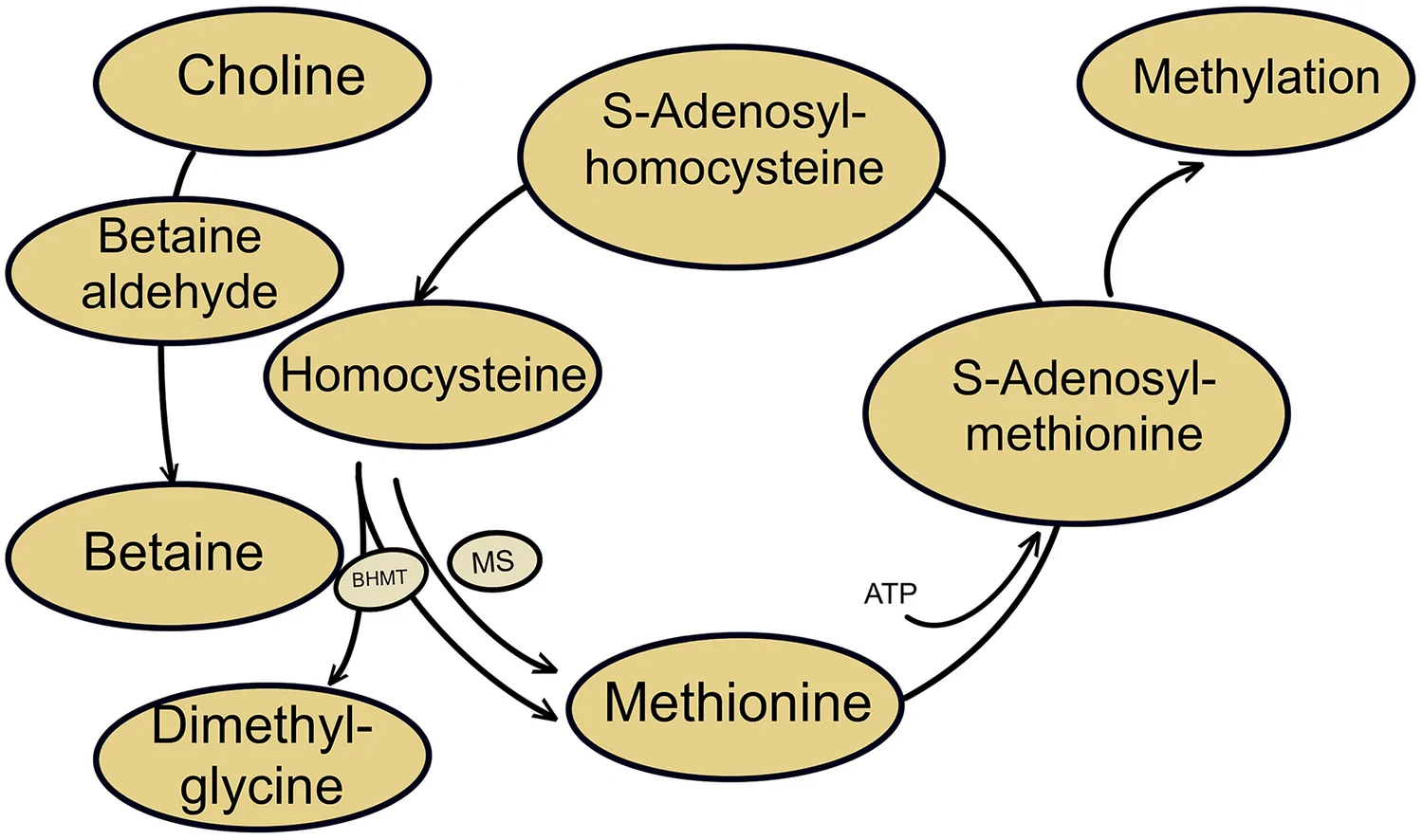
Role of betaine in the methionine cycle in liver. Betaine provides an alternative pathway for methylation of homocysteine. *BHMT* Betaine-homocysteine S-methyltransferase, *MS* Methionine synthase. [21] Adapted from Craig et al. and modified by the authors

Following peak detection, alignment, and quality filtering, a total of approximately 1200 reproducible features were retained for statistical analysis. Unsupervised hierarchical clustering of the normalized feature matrix, including pooled QC samples, confirmed that group-wise differences in metabolomic profiles. Following mass spectrometry data processing and metabolite identification, fold change analysis labeled significantly altered metabolites between groups. Pathway analysis linked these metabolites to several pathways, including amino acid and pyrimidine metabolism, and ABC transporters (Figs. 5 and 6). Betaine levels between Groups A and B did not exhibit a statistically significant difference. However, the fold change (FC) of 2.74 suggests some degree of increase. In contrast, a notable difference was observed between Groups A and C (*p* = 0.0242), with a total score of 0.88 and an FC value of 0.36, indicating a decrease in betaine levels compared to the control group. Proline levels were also elevated significantly between the Groups A and C (*p* = 0.03), showed by an FC level of 1.4 and total score of 0.85. However, proline was absent from the A vs. B dataset, suggesting that it was not detected or did not meet the threshold for inclusion among identified metabolites. Elevated proline levels between the Groups A and C contributed to the upregulation of the arginine and proline metabolism pathway (Figs. 6 and 7). Although no significant differences in arginine levels were observed between the groups, the pathway was still upregulated when comparing Groups A and C. Changes in proline and betaine levels are shown in Table 2 (Fig. 8).

**Table 2:** Comparison of betaine and proline levels between groups based on LC-MS analysis

Groups	Metabolite	*p*-value	Total score	Fold change (FC)
A vs. B	Betaine	> 0.05	0.88	2.70
A vs. B	Proline	–	–	–
A vs. C	Betaine	**0.024**	0.88	0.367
A vs. C	Proline	**0.03**	0.85	1.40

Proline was not detected in the Group A vs. Group B comparison. Fold change (FC) < 1 indicates a decrease relative to Group A; FC > 1 indicates an increase

Bold values highlight the significant *p* value

### EMG results

**Fig. 9:**
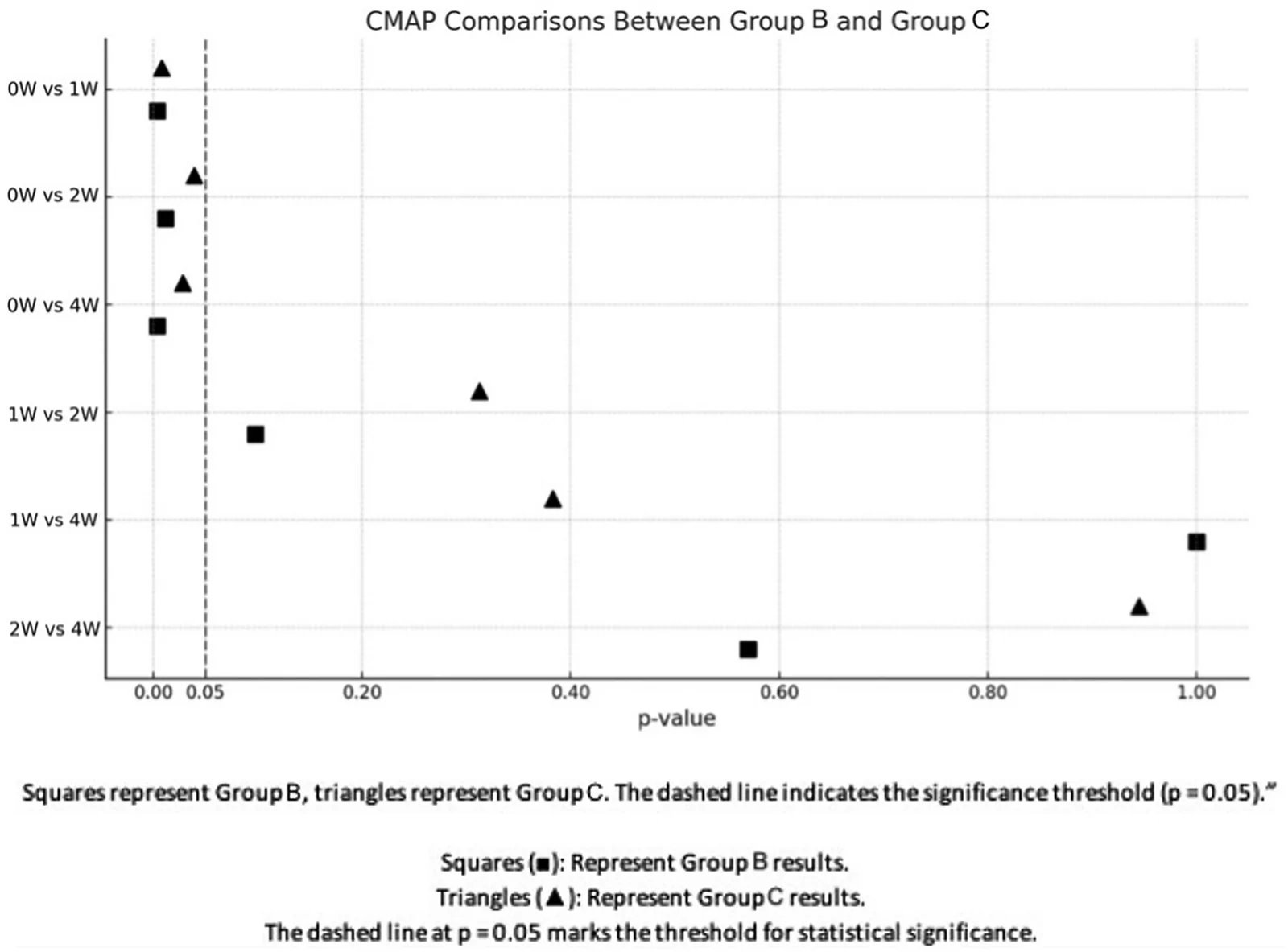
CMAP comparisons between group B and group C

The effects of minocycline on nerve regeneration were assessed by analyzing CMAP and DML values over time. One-way ANOVA results showed no statistically significant differences in CMAP values among the groups at week 1 (*p* = 0.66) and week 4 (*p* = 0.56) (Table 3). Similarly, DML values did not significantly differ between groups at week 1 (*p* = 0.50) and week 4 (*p* = 0.89) (Table 4). Repeated measures ANOVA revealed a significant time effect (*F*(3, 69) = 22.31) (*p* < 0.05, η^2^ = 0.492) (Table 5). However, no significant group effect (*F*(2, 23) = 1.133) (*p* > 0.05) or time × group interaction (*F*(6, 69) = 2.013) (*p* > 0.05) was detected. The mean ± standard deviation (SD) CMAP values across groups and time points are provided in Table 6. The Friedman test indicated significant within-group differences in Group B (*χ*^*2* ^= 14.20, *p* = 0.013) (Table 7). In Group C, less pronounced changes were observed (*χ*^*2*^ = 8.47) (*p* = 0.037). Group A did not show significant changes in CMAP values over time (*χ*^*2*^ = 5.9), (*p* = 0.165). The Wilcoxon signed-rank test revealed significant differences between baseline (0W) and week 2 (2W) (*p* < 0.05) (Table 8). These differences remained significant after Bonferroni correction. No significant differences in DML values were detected across groups or over time. Figure 9a presents the statistical comparisons of CMAP values between Group B and Group C. Significant differences were observed at 0W vs. 1W, 0W vs. 2W, and 0W vs. 4W, whereas comparisons at 1W vs. 2W, 1W vs. 4W, and 2W vs. 4W were not statistically significant. The post hoc pairwise comparisons (Table 9) demonstrated that significant improvements in CMAP values were primarily observed between baseline (0W) and later time points in Group B, with the most pronounced changes occurring in week 2 (2W) and week 4 (4W). In contrast, Group C exhibited less consistent changes, with only minor improvements observed between 0W vs. 2W and 0W vs. 4W. These findings suggest that Group B contributed more effectively to CMAP recovery over time compared to Group C. However, no significant differences were detected at later stages, indicating a plateau in CMAP improvements after week 2.

**Table 3:** One-way ANOVA results for CMAP

Group	CMAP (Week 1)	CMAP (Week 4)
A	4.29 ± 2.02 mV	4.38 ± 2.64 mV
B	3.63 ± 1.55 mV	3.69 ± 3.16 mV
C	4.12 ± 0.84 mV	5.29 ± 3.35 mV
*p*-value	0.66	0.56

This table shows the mean CMAP values (± SD) on the right facial nerve at weeks 1 and 4 post-surgery. (*P* < 0 .05 is considered statistically significant)

**Table 4:** One-way ANOVA results for DML

Group	DML (Week 1)	DML (Week 4)
A	1.26 ± 0.35 ms	1.32 ± 0.49 ms
B	1.28 ± 0.35 ms	1.27 ± 0.30 ms
C	1.45 ± 0.39 ms	1.24 ± 0.35 ms
*p*-value	0.5 *	0.89**

(*) One-way ANOVA was used for these variables. (**) Welch’s ANOVA was applied due to violation of homogeneity (Levene’s test, *p* < 0.05). (*p* < 0 .05 is considered statistically significant)

Bold values highlight the significant *p* value

**Table 5:** Repeated measures ANOVA results for CMAP

Effect	*F* (d*f*1, d*f*2)	*p*-value	*η*^2^
Time effect	*F* (3, 69) =22.31	< 0.05	0.492
Group effect	*F* (2, 23) =1.133	0.339	0.09
Time × group interaction	*F* (6, 69) =2.013	0.076	0.149

F *(d*f*1, d*f*2): F-*statistic with degree of freedom *p* < 0.05 is considered statistically significant. Partial eta squared (*η*^2^): Indicates effect size (0.01 = small, 0.06 = medium, 0.14 = large)

**Table 6:** CMAP values across groups and time points

Time point	Mean ± SD (group B)	Mean ± SD (group C)
Baseline (0W)	4.33 ± 1.34	4.82 ± 1.57
Week 1 (1W)	3.46 ± 0.98	3.99 ± 1.51
Week 2 (2W)	3.16 ± 1.06	4.23 ± 1.50
Week 4 (4W)	3.47 ± 1.21	4.30 ± 1.75

**Table 7:** Pairwise comparison of CMAP values across time points

Group	Friedman test *χ*^2^	*p*-value	Kendall’s *W*
A	5.9	0.165	0.17
B	14.20	**0.013**	0.47
C	8.47	**0.037**	0.29

Bold values highlight the significant *p* value

**Table 8:** Wilcoxon signed-rank test for DML pairwise comparisons

Comparison	*Z*	*p*-value	Bonferroni correction
Baseline vs. week 1	6	0.1763	1
Baseline vs. week 2	3	**< 0.05**	0.2128
Baseline vs. week 4	10	0.1641	0.9844
Week 1 vs. week 2	10	0.2614	1
Week 1 vs. week 4	13.5	0.5276	1
Week 2 vs. week 4	12.5	0.7995	1

Bold values highlight the significant *p* value

**Table 9:** Wilcoxon signed-rank test for CMAP pairwise comparisons

Comparison	*Z*	*p*-value	Bonferroni correction
Baseline vs. week 1	7	0.977	0.5859
Baseline vs. week 2	1	**< 0.05**	0.0469
Baseline vs. week 4	6	0.929	0.5574
Week 1 vs. week 2	10	0.641	0.9844
Week 1 vs. week 4	18.5	0.6523	1
Week 2 vs. week 4	13.5	0.3008	1

Bold values highlight the significant *p* value

## Discussion

Peripheral nerve damage (PNI) remains a major clinical problem. According to an English study, there has been a 57% rise in hand trauma occurrences, which primarily impact men and people between the ages of 15 and 59. Higher rates of fractures and nerve damage have been linked to this increase [21]. The increasing prevalence of PNI reflects growing public health concern and underscores the relevance of the present study.

Minocycline is a second-generation tetracycline known mainly for its antimicrobial activity in bacterial infections [6]. However, minocycline has recently gained attention for its non-antibiotic properties, including anti-inflammatory [7], tumor suppressive [8], and neuroprotective effects [9]. Studies have shown that the local application of minocycline promotes sciatic nerve regeneration after injury by activating Schwann cells and modulating key cell marker expressions essential for successful regeneration, thereby improving functional outcomes while avoiding systemic side effects [1]. In the present study, we investigated the effects of locally applied minocycline on facial nerve regeneration in rats. To our knowledge, this is the first study reporting the beneficial effects of local minocycline on facial nerve regeneration, as supported by histological, electrophysiological, and LC-MS analyses. In general, tetracyclines have shown promise in various neurological disorder models, such as stroke [10], spinal cord injury [11], Parkinson’s disease [12], and Huntington’s disease [13] by inhibiting inflammatory cytokines and protecting neural tissue. Minocycline’s effect on Alzheimer’s disease has also been documented, concluding that minocycline suppressed microglial activity, thereby reducing cytokine production and improving cognitive performance in rats [14].

The local application of minocycline in our study helps eliminate the risk of systemic side effects that have been reported with oral minocycline use. Among these, hepatic complications such as irreversible drug-induced hepatitis and fulminant hepatic failure have been documented. Other reported adverse effects include intracranial hypertension and vision loss [15].

In a study investigating facial nerve regeneration in rats, the experimental period was set at 25 days, during which clinical findings were monitored. It was reported that rats in the treatment group demonstrated varying degrees of functional improvement in the blink reflex and vibrissae movement compared to crush-only group [16]. In our study, the clinical findings showed similar results to this study, indicating that minocycline consistently promotes recovery in models of facial nerve injury. Moreover, in addition to the functional parameters evaluated in the previous studies, the inclusion of apex nasi deviation expanded the scope of clinical assessment in our study. Notably, functional improvements were more pronounced in Group B, particularly in terms of clinical findings, suggesting that minocycline may exhibit a dose–response relationship. Furthermore, these results highlight the importance of dose titration in determining optimal therapeutic efficacy. In this context, the present study makes a meaningful contribution to the literature by both broadening the methodological framework and providing novel data regarding the dose sensitivity of minocycline.

In the present study, the most obvious findings due to nerve damage were edema and vacuolization. Despite the nerve fiber density did not differ significantly between groups, minocycline treatment at both doses showed beneficial effect on those changes. Although any microscopic studies have not reported the effects of minocycline on fascial nerve regeneration, Sun et al. [32] have reported minocycline’s alleviating ability on retinal edema in the early stages of branch retinal vein occlusion in hematoxylin–eosin-stained sections, partly by inhibiting TNF-α expression. Another histological study has reported beneficial effects of minocycline on decreasing percentage of vacuolation in the white matter after spinal cord injury, partly due to an inhibition of microglial activation [17].

Untargeted metabolomics analysis conducted to identify differences in metabolite profiles and affected metabolic pathways among groups revealed abnormalities in betaine and proline metabolisms. Betaine is an alpha-amino acid that plays a significant role in the methionine cycle, a part of the methionine metabolic pathway. Through the enzyme betaine-homocysteine methyltransferase, it facilitates the re-methylation of homocysteine to methionine [18–22]. Betaine plays multiple biological roles; for example, in Apc-mutant mice, a methyl donor-deficient (MDD) diet reduced betaine levels on LC-MS/MS analysis, suggesting metabolic changes relevant to colorectal cancer prevention [23]. In our study, betaine levels in Group C were significantly lower than controls. This parallels the decrease reported under an MDD diet, suggesting that betaine deficiency may be linked to the nerve regeneration effects seen in Group C. Group B showed a non-significant increase, possibly reflecting a compensatory metabolic response to minocycline, rather than direct regenerative effect. Proline is an amino acid involved in protein synthesis [24] and oxidative stress regulation [25]. One study investigated the role of Sox11 in enhancing nerve regeneration in mice with saphenous nerve injury. Findings suggest that Sox11 promotes nerve regeneration through the activation of small proline-rich protein 1a (Sprr1a). Overexpression of Sox11 led to increased neurite outgrowth in neurons and enhanced axon regeneration, thereby improving their functional recovery [26]. Our LC-MS/MS results demonstrated a significant increase in proline levels only in Group C. However, proline was absent in Group B. Pathway analysis also showed that minocycline significantly enhanced the arginine–proline metabolism. Although arginine levels were not significantly different, the pathway remained upregulated in Group C compared to controls.

Our EMG findings reveal that Group B experienced notable improvements in CMAP and DML values. This aligns with the previous studies reporting similar outcomes [1, 27,28]. The observed improvements suggest that minocycline may enhance axonal integrity and promote remyelination processes. Interestingly, the increased dose in Group C did not yield additional benefits, suggesting a plateau effect in the dose–response relationship. One hypothesis for this observation is that higher doses may trigger compensatory mechanisms, such as anti-inflammatory saturation or cellular toxicity thresholds, which could dampen further neuroprotective effects [1, 29]. Minocycline’s neuroprotective pathways may reach maximum efficacy at moderate doses, beyond which additional effects are negligible or even counterproductive. Another key finding of our study is the significant role of time in the recovery process. Repeated measures analysis highlighted that healing is a dynamic process, becoming more pronounced over time rather than being strictly dose dependent. This emphasizes that the beneficial effects of minocycline on nerve recovery may continue to evolve, and longer follow-up periods could reveal more pronounced differences between treatment groups. Longitudinal studies are warranted to further explore its sustained neuroregenerative potential and impact on long-term functional outcomes. Our results are consistent with those of Açıkgöz Yıldız et al. [30], who demonstrated that neurotrophic agents such as IGF-1 and PRP positively influence nerve regeneration. Similarly, Longur *et al.* [31] reported that bumetanide, particularly when combined with dexamethasone, enhances nerve recovery post-injury. These studies underscore the potential of pharmacological interventions to modulate nerve healing pathways and highlight the value of exploring combination therapies to maximize recovery. Studies by Fei et al. [32], Heaton et al. [33], and Henstrom et al. [34] further supported the relevance of our electrophysiological assessments, providing critical insights into the anatomical and functional recovery of facial nerves. Their work complements our findings, emphasizing the value of precise anatomical understanding in optimizing treatment strategies for facial nerve injuries. Importantly, minocycline’s affordability and widespread availability make it an attractive candidate for clinical application. Its well-documented neuroprotective effects on peripheral nerves are now corroborated in the context of facial nerve injuries, bridging a crucial gap in translational research. This suggests that minocycline could be readily integrated into clinical practice, especially in resource-limited settings where cost-effective treatments are essential. Further studies are warranted to confirm its efficacy in diverse clinical populations and to establish standardized dosing protocols. The electrophysiological methodologies employed in our study are supported by nerve conduction models described by Salomone et al. [35] and Takeuchi et al. [36] ensuring the reliability and reproducibility of our CMAP and DML measurements. These standardized methods enhance the comparability of our findings with existing literature and support the validity of our conclusions. In conclusion, moderate doses of minocycline effectively promote nerve regeneration, whereas higher doses offer no added benefit, likely due to a plateau in neuroprotective effects. Dose optimization, long-term evaluation, studies on synergistic therapies, and further elucidation of its mechanisms of action are warranted [27–29, 37,38]. Additionally, clinical trials are needed to confirm its applicability in peripheral nerve injuries.

## Conclusions

In this study, we comprehensively evaluated facial nerve regeneration at molecular, cellular, clinical, and functional levels, integrating LC-MS-based metabolomics, histological analyses, and EMG testing. This is the first study to apply LC-MS analysis to assess metabolic changes following facial nerve injury, filling an important gap in the literature on the metabolic changes of peripheral nerve regeneration. Our findings indicate that while Group C yielded superior histological and biochemical outcomes, Group B resulted in improved clinical and electrophysiological outcomes; this suggests dose-dependent differences in the regenerative effects of minocycline compared to the control group and indicates that fibrin gel has no effect on nerve regeneration. The strength of our work lies in its multi-level approach and deep metabolomic insights. However, the potential of betaine and proline metabolites as biomarkers in the context of minocycline-induced nerve regeneration remains to be validated in detailed and longer-term studies.

## Limitations

This study presents with limitations to be acknowledged. While LC-MS revealed important metabolic changes, additional techniques such as Nuclear Magnetic Resonance (NMR) spectroscopy and electron microscopy could be utilized to better characterize the molecular pathways involved in nerve regeneration through gene or protein expression assays. In addition, the relatively small sample size may have limited the statistical power of our analysis.

## Data Availability

The datasets generated and analyzed during this study are available from the corresponding author upon reasonable request.
