# Correction: Dose-Dependent Effects of Minocycline on Facial Nerve Regeneration: A Microscopic and Molecular Study

**DOI:** 10.1007/s00266-026-06214-2

**Published:** 2026-08-20

**Authors:** Sumera İnci Yılmaz, Sedat Meydan, Mukaddes Eşrefoğlu, Nihat Mustafayev, Leyla Mustafayeva, Kübra Başol Baki, Metin Demirel, Fatih Maral, Şahabettin Selek

**Affiliations:** 1 Faculty of Medicine, Bezmialem Vakıf University, Istanbul, Turkey; 2 Faculty of Medicine, Department of Anatomy, Bezmialem Vakıf University, Istanbul, Turkey; 3 Faculty of Medicine, Department of Histology and Embryology, Bezmialem Vakıf University, Istanbul, Turkey; 4 Nihat Mustafayev Clinic, Istanbul, Turkey; 5 Faculty of Medicine, Department of Physical Therapy and Rehabilitation, Cerrahpaşa Istanbul University, Istanbul, Turkey; 6 Faculty of Medicine, Department of Medical Biochemistry, Bezmialem Vakıf University, Istanbul, Turkey; 7 Faculty of Medicine, Department of Anatomy, Marmara University, Istanbul, Turkey; 8 Department of Medical Services and Techniques, Vocational School of Health Services, Pathology Laboratory Techniques Program, Bezmialem Vakıf University, Istanbul, Turkey

**Correction to: Aesth Plast Surg** **https://doi.org/10.1007/s00266-026-05976-z**

The original online version of this article was revised to denote that coauthor Sedat Meydan is the primary corresponding author.

The original article has been corrected.

